# On the importance of understanding physiology when estimating energetics in cetaceans

**DOI:** 10.1242/bio.023929

**Published:** 2017-02-08

**Authors:** Lars P. Folkow, Arnoldus Schytte Blix

**Affiliations:** 1 Department of Arctic and Marine Biology, UiT – The Arctic University of Norway, Tromsø NO-9037, Norway; 2 St. Catharine's College, Cambridge CB2 1RL, UK

## Abstract

**Summary:** Fahlman and associates (2016) have emphasized the importance of proper physiological insight when modelling energy expenditure in large cetaceans. Here we argue that they have themselves failed in this endeavour.

We have, with great interest, read the paper entitled ‘Estimating energetics in cetaceans from respiratory frequency: why we need to understand physiology’ by Fahlman et al. (2016), hereafter referred to as Fahlman et al., and we are particularly pleased by their emphasis of the need to understand physiology when estimating field metabolic rates (FMR) of cetaceans.

In this paper, Fahlman et al. have employed their own detailed data collected from captive bottlenose dolphins (*Tursiops truncatus*), at rest and at rest after a swimming bout at surface, to test the validity of using respiratory variables of free-living large mysticete cetaceans for the estimation of their FMR. This approach was first used in the blue whale (*Balaenoptera musculus*) by Nobel laureate August Krogh, who definitely knew physiology (Krogh, 1934), and has hitherto been the only way to obtain useful estimates of energy expenditure in free-living large cetaceans, which are not easily accommodated in laboratories. It takes advantage of the fact that diving mammals are breath-hold divers which benefit from maximizing their time under water by conducting respiratory exchange during brief surface periods, in which large volumes of air are rapidly exchanged (e.g. Blix and Folkow, 1995).

The approach is based on the equation:(1)
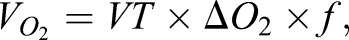
where *V*_*O*_2__ is the rate of oxygen uptake (l min^−1^); *VT* is tidal volume (l breath^−1^); Δ*O_2_* is the difference in oxygen concentration between inspired (*O_2in_*) and expired air (*O_2ex_*); Δ*O_2_=O_2in_−O_2ex_* (%); and *f* is breathing rate (breaths min^−1^).

In their analysis, Fahlman et al. have evaluated three methods, all based on Eqn 1 (Methods A, B and C). In Method A, data from Armstrong and Siegfried (1991) and Dolphin (1987) were used, and in B our data (Blix and Folkow, 1995) were used, in both cases to estimate FMR in large mysticetes; whereas in Method C, Fahlman et al. used their own data from their dolphins. In all cases the method outputs were compared to Fahlman et al.'s own direct measurements of *V*_*O*_2__ in the same dolphins. In so doing it is hardly surprising that Method C performed well, whereas Methods A and B were found to overestimate the ‘true’ value by some 200-500% and, although Fahlman et al. offer some reservations on the validity of their comparison, this remains to be the take-home message, both from the abstract and the full paper.

However, we want to emphasize that Fahlman et al. conducted their comparison across levels of activity, across suborders and across >20-orders-of-magnitude in body mass, and in doing so, even chose to use empirical allometric relationships for prediction of lung volumes in large mysticetes to predict the lung volumes of their dolphins, instead of using their own measured values. That the outputs of the different methods differ substantially should therefore come as no bigger surprise than that use of the Du Bois formula for estimation of surface area in man (Du Bois and Du Bois, 1916) would prove inappropriate for estimating surface area, for example, in giraffes. Moreover, if Fahlman et al. instead had estimated the lung volumes of their dolphins by use of Wahrenbrock and coworker's (1974) empirical equation for gray whales (*Eschrichtius robustus*), they would have obtained negative values.

To further illustrate our point, we have tested the validity of Fahlman et al.'s Method C, using existing data for a large mysticetes. Wahrenbrock et al. (1974) have measured that a 4000 kg gray whale calf at rest has a *V*_*O*_2__ corresponding to about 2× Kleiber's basal metabolic rate (BMR), i.e. as expected for any immature and growing mammal (Kleiber, 1961). Using Method C with Fahlman et al.'s data for resting *VT* (32% of total lung capacity) and Δ*O_2_* (4.94%), and Wahrenbrock and coworker's (1974) measured values of lung volume (236 liter) and resting breathing rate (∼1 breath min^−1^) for a calf of 4000 kg, we obtain a *V*_*O*_2__ of 3.7 liter min^−1^, which is less than 35% of the *V*_*O*_2__ of 10.7 liter min^−1^ that was measured. If Fahlman's Method C is applied to our data (Blix and Folkow, 1995), it suggests that a minke whale (*Balaenoptera acutorostrata*) would be able to swim at 2–3 m s^−1^ at a FMR that is 20% below Kleiber's BMR of mammals in general (Kleiber, 1961), or for good measure, at similar FMR (in W kg^−0.75^) as in the three-toed sloth (*Bradypus variegatus*) (Nagy and Montgomery, 1980).

One would assume that, with proper physiological insight, the extraordinarily large discrepancies between the outcomes of the different methods would have inspired similar exercises, and that Fahlman et al.'s paper, in consequence, would not have been published in its present form.
